# Adipose Tissue Denervation Blunted the Decrease in Bone Formation Promoted by Obesity in Rats

**DOI:** 10.3390/nu15163574

**Published:** 2023-08-14

**Authors:** Milene Subtil Ormanji, Maria Victória Lazarini Melo, Renata Meca, Michelle Louvaes Garcia, Ana Carolina Anauate, Juan José Augusto Moyano Muñoz, Lila Missae Oyama, Erika Emy Nishi, Cassia Toledo Bergamaschi, Aluizio Barbosa Carvalho, Ita Pfeferman Heilberg

**Affiliations:** 1 Nephrology Division, Universidade Federal de São Paulo, São Paulo 04023-062, Brazil; msormanji@unifesp.br (M.S.O.); mavi.lazarini@hotmail.com (M.V.L.M.); rmeca28@gmail.com (R.M.); anauatte@gmail.com (A.C.A.); juanjomoy@gmail.com (J.J.A.M.M.); aluizio@uol.com.br (A.B.C.); 2 Department of Physiology, Universidade Federal de São Paulo, São Paulo 04023-062, Brazil; michelle.louvaes@gmail.com (M.L.G.); lmoyama@unifesp.br (L.M.O.); enishi@unifesp.br (E.E.N.); bergamaschi.cassia@unifesp.br (C.T.B.)

**Keywords:** bone histomorphometry, bone–nervous system interactions, bone–fat, neuropeptide Y

## Abstract

The impact of obesity upon bone metabolism is controversial since both beneficial or harmful effects have been reported. Bone remodeling is modulated by the central nervous system through cytokines, hormones and neuromodulators. The present study aimed to evaluate the effects evoked by bilateral retroperitoneal white adipose tissue (rWAT) denervation (Dnx) upon bone mineral metabolism and remodeling in an experimental model of obesity in rats. Male Wistar rats were fed during 18 weeks with high-fat diet (HFD) or standard diet (SD) as controls, and rWAT Dnx or Sham surgery was performed at the 14th week. Biochemical and hormonal parameters, bone histomorphometry, rWAT and hypothalamus protein and gene expression were analyzed. The HFD group presented decreased bone formation parameters, increased serum and bone leptin and FGF23, increased serum and hypothalamic neuropeptide Y (NPY) and decreased serum 1,25-dihydroxyvitamin D_3_ and PTH. After rWAT Dnx, bone markers and histomorphometry showed restoration of bone formation, and serum and hypothalamic NPY decreased, without alteration in leptin levels. The present study shows that the denervation of rWAT improved bone formation in obese rats mediated by a preferential reduction in neurohormonal actions of NPY, emphasizing the relevance of the adipose tissue–brain–bone axis in the control of bone metabolism in obesity.

## 1. Introduction

Obesity predisposes to several comorbidities, but its interaction effects in bone metabolism are complex and not yet fully elucidated. Although increased body weight has traditionally been considered as protective against osteoporosis due to mechanical loading [1,2,3], more recent studies have reported an inverse association of fat mass with bone mineral density (BMD) in both women and men [4,5,6] and with bone formation by histomorphometric analysis [7]. Experimentally, a high-fat diet (HFD) is known to induce “whitening” of the brown adipose tissue, which possesses bone anabolic effects [8,9]. However, the underlying mechanisms behind this controversy remain unknown.

A recent meta-analysis evidenced that both obesity and overweight are characterized by sympathetic overactivation to different target organs, contributing to the development of comorbidities, such as hypertension and increased risk of cardiovascular disease [10]. Interestingly, some epidemiological studies have disclosed that the use of beta-adrenergic blocking therapy for hypertension was associated with increased BMD and decreased hip fracture risk, suggesting that the central control of bone mass is mediated by a neuronal pathway involving the sympathetic nervous system [11,12]. Moreover, treatment with propranolol, a nonselective beta-adrenergic blocker, significantly increases bone formation and bone mass in mice [13]. Adipokines and hormones released by adipose tissue such as leptin and others have complex effects on bone metabolism, as demonstrated by several experimental studies [14,15]. Although it has been suggested that leptin may exert direct stimulatory effects on bone cells as opposed to indirect inhibitory brain effects [16,17], it is still a matter of controversy [14]. Leptin receptor ObRb is expressed in serotonergic neurons within the brainstem that project to the ventromedial hypothalamus, where they control bone mass through hypothalamic-generated sympathetic tone. Increased sympathetic drive mediates signaling in osteoblasts through β2-adrenergic receptors inhibiting bone formation and increasing release of the receptor activator of nuclear factor kappa-Β ligand (RANKL) thus increasing bone resorption [13,18]. Neuropeptide Y (NPY) is another neuromodulator of bone metabolism, widely distributed in the central nervous system (CNS), but mainly expressed in the hypothalamus and released from the sympathetic nerve terminal [19,20,21]. It acts predominantly through its receptors Y1, expressed in peripheral tissues including osteoblasts [22], and Y2, expressed predominantly in the hypothalamus [23]. The underlying mechanism of NPY action in bone mass may involve the inhibition of the cAMP pathway followed by ERK phosphorylation, resulting in osteoblast differentiation inhibition [24] which in turn is seen in mouse models expressing higher levels of NPY [25]. A negative effect on bone mass has been described under conditions of NPY excess [26].

Given that retroperitoneal white adipose tissue (rWAT) is an important fat visceral depot which receives intense sympathetic and sensory innervation, and that its denervation is able to induce systemic responses in other organs and tissues as previously reported [27], the present study aimed to determine the effects evoked by bilateral rWAT denervation (Dnx) on the adipose tissue–CNS–bone axis. We evaluated biochemical and hormonal parameters of bone mineral metabolism, including leptin and NPY, as well as bone histomorphometry, in an experimental model of high-fat diet (HFD)-induced obesity in rats before and after rWAT Dnx.

## 2. Materials and Methods

### 2.1. Animals

**Figure 1:**
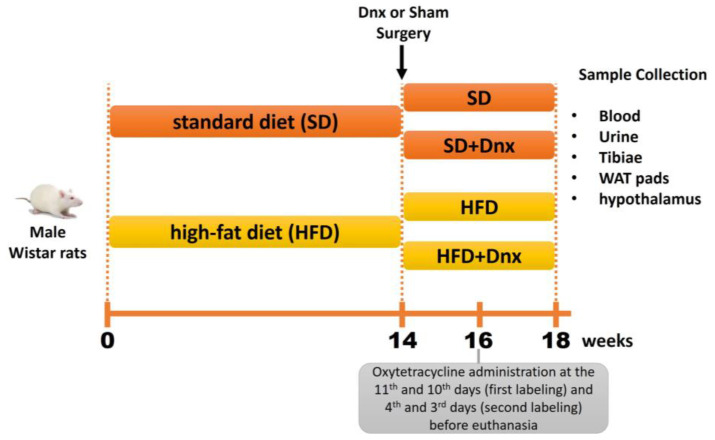
Schematic figure of the experimental design. Dnx—denervation surgery; WAT—White Adipose Tissue.

Male Wistar rats (9 weeks old) housed under controlled environmental conditions (25 °C, on a 12 h light/dark cycle) were given a high-fat diet (HFD) or standard diet (SD) as controls for a period of 18 weeks, and body weight (BW) was recorded weekly (Figure 1). Rats were injected intraperitoneally with 20 mg/kg oxytetracycline (Pfizer, Manhattan, New York, NY, USA), at the 11th and 10th days (first labeling) and 4th and 3rd days (second labeling) before euthanasia for kinetic evaluation of bone histomorphometry. Twenty-four urine samples were collected at the 18th week followed by euthanasia of animals by an intraperitoneal anesthetic overdose of ketamine and xylazine (270 and 90 mg/kg i.p., respectively). Fasting blood was collected via cardiac puncture and stored at −80 °C for biochemical and hormonal analyses. Both tibiae, rWAT and hypothalamus were removed and stored for bone histomorphometry, protein and gene expression analyses. The study was approved by the Ethics Committee for Animal Experiments of the University (No. 3561110817/2017) and was reported in agreement with ARRIVE guidelines (https://arriveguidelines.org, accessed on 11 September 2017). All experiments were performed in accordance with relevant guidelines and regulations.

### 2.2. Diet Composition

The SD diet contained 9 Kcal% of fat, 15 Kcal% of protein and 76 Kcal% of carbohydrate, providing 3.80 Kcal/g (American Institute of Nutrition—AIN-93) [28]. HFD was modified from AIN-93 (Supplementary Table S1) by adding lard to provide 60 Kcal% of fat, 15 Kcal% of protein and 25 Kcal% of carbohydrate, yielding 5.36 Kcal/g. Micronutrient content from both diets were equivalent.

### 2.3. Surgery

As shown in Figure 1, total bilateral rWAT denervation (Dnx) or Sham surgery were performed at the 14th week of the protocol under ketamine and xylazine (80 and 10 mg/kg, i.p., respectively) anesthesia.

**Figure 2:**
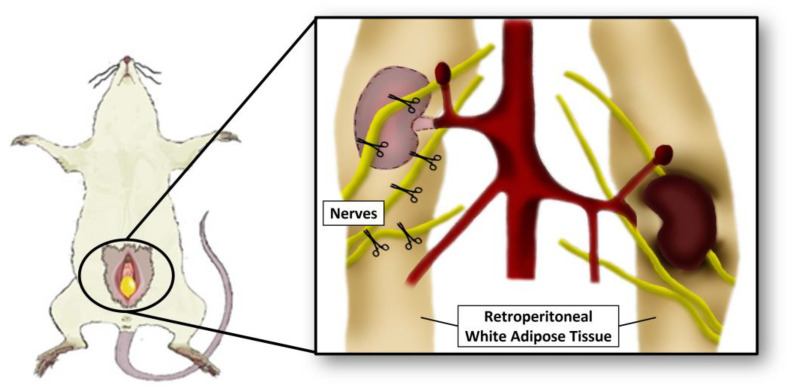
Anatomical localization scheme of sympathetic nerves sectioned at denervation surgery (Dnx).

The sympathetic nerves to the retroperitoneal WAT were sectioned before and after crossing the fat pad and a portion of approximately 2 mm of each nerve was removed (Figure 2) [29]. In Sham surgery, the three branches innervating the rWAT were identified but not sectioned. As postoperative analgesia, meloxicam was administered for 3 days (1 mg/kg/day, i.m.).

### 2.4. Confirmation of rWAT Denervation by Western Blot

The proteins were extracted from rWAT using RIPA solution and quantified using the modified Lowry method (Bio-Rad laboratories, Hercules, CA, USA). Proteins (30 μg) were separated by 10% SDS-PAGE electrophoresis, electro-blotted to nitrocellulose membranes (GE Life Sciences, Bracknell, UK) and incubated with primary and secondary antibodies as described in Supplementary Table S2. Immobilon Western HRP substrate (Millipore, MA, USA) was used to visualize protein bands and Uvitec analysis software (Uvitec Limited, Cambridge, UK) for quantification.

### 2.5. Serum Biochemical Markers and Hormones

Serum and urinary calcium, phosphorus and creatinine were measured using colorimetric assays (Labtest Diagnostics, Lagoa Santa, Brazil). The fractional excretion of phosphorus (FEP) and fractional excretion of calcium (FECa) were calculated as FEP = [(urinary phosphorus × serum creatinine)/(serum phosphorus × urinary creatinine)] × 100 and FECa = [(urinary calcium × serum creatinine)/(serum calcium × urinary creatinine)] × 100. Levels of 25-hydroxyvitamin D_3_ (25(OH)D_3_) and 1,25-dihydroxyvitamin D_3_ (1,25(OH)_2_D_3_) were determined using a chemiluminescence immunoassay kit (LIAISON^®^ Diasorin Inc, Stillwater, OK, USA). A Milliplex rat bone panel (Millipore, Billerica, MA, USA; Cat# RBN1MAG-31K) was used to measure serum levels of parathyroid hormone (PTH), fibroblast growth factor 23 (FGF23), sclerostin and Dickkopf related protein 1 (DKK1) using Luminex magnetic microbead array technology. Specific ELISA Kits were used to determine serum levels of leptin, NPY (Millipore, MA, USA and MyBiosource, San Diego, CA, USA, respectively), procollagen type I amino-terminal propeptide (P1NP) and C-terminal telopeptide of type I collagen (CTX), respectively (Immunodiagnostic Systems, Boldon, UK).

### 2.6. Bone Histomorphometry

The right tibia was removed and embedded in methyl methacrylate as previously described [30]. Bone sections of 5 μm were cut and stained with toluidine blue. The bone histomorphometric parameters were measured at the region of the proximal metaphysis, 195 μm from the epiphyseal growth plate, using a semi-automatic image analyzer (Osteomeasure; Osteometrics Inc., Atlanta, GA, USA). Static parameters included the bone volume (BV/TV, %), trabecular number (Tb.N, n/mm), trabecular thickness (Tb.Th, μm), trabecular separation (Tb.Sp, μm), osteoid volume (OV/BV, %), osteoid thickness (O.Th, μm), osteoblastic and osteoid surface (Ob.S/BS and OS/BS, %, respectively), osteoclastic and eroded surface (Oc.S/BS and ES/BS, %, respectively). Kinetic parameters, obtained from unstained 10 μm sections and evaluated under ultraviolet light microscopy, included the mineralizing surface (MS/BS, %), mineral apposition rate (MAR, μm/day), bone formation rate (BFR/BS, μm^3^/μm^2^/day) and mineralization lag time (Mlt, days). All animal data were obtained by blind measurements and all histomorphometric parameters were reported according to the standardized nomenclature recommended by the American Society of Bone and Mineral Research [31]. The histological analysis was performed in the Laboratory of Renal Osteodystrophy at the Universidade Federal de São Paulo.

### 2.7. Protein Expression Analysis

The bone marrow of the tibia was removed by placing the tibia with the cut end down in a centrifuge tube followed by centrifugation for 30 s at 5700× *g*. The hypothalamus was collected as previously described [32]. Proteins were extracted with Trizol reagent (Thermo Scientific, Waltham, MA, USA) and quantified using the modified Lowry method (Bio-Rad laboratories, Hercules, CA, USA). Bone protein samples were used to perform Milliplex rat bone panel (Millipore, Billerica, MA, USA; Cat# RBN1MAG-31K) to determine bone protein levels of leptin, FGF23, sclerostin and DKK1 by Luminex magnetic microbead array technology and hypothalamus protein samples were used to determine NPY levels by specific Elisa Kit (MyBiosource, San Diego, CA, USA).

### 2.8. RNA Extraction and Real Time PCR

The hypothalamic mRNA was extracted using Trizol (Thermo Scientific, MA, USA) and reverse-transcribed using a High-Capacity cDNA Reverse Transcription Kit (Thermo Scientific, MA, USA). Quantitative real-time PCR was performed in QuantStudio^®^ 7 Flex real-time PCR system (Thermo Scientific, MA, USA) with specific primers (Supplementary Table S3). The relative mRNA levels were calculated as 2^(−ΔΔCt)^ method. HPRT was used for normalization of gene expression.

### 2.9. Statistical Analysis

Group mean values were compared using two-way Analysis of Variance (ANOVA) followed by Bonferroni post hoc test whenever a significant interaction was detected. Variables non-normally distributed were log-transformed to stabilize variance. *p* Values < 0.05 were considered statistically significant. SPSS v.23 (IMB Inc., Armonk, NY, USA) and GraphPad Prism v.8 (GraphPad Software, San Diego, CA, USA) were used for calculations and graphics.

## 3. Results

### 3.1. Body Weight (BW), Waist Circumference (WC) and White Adipose Tissue (WAT) Pads before and after Dnx

**Figure 3:**
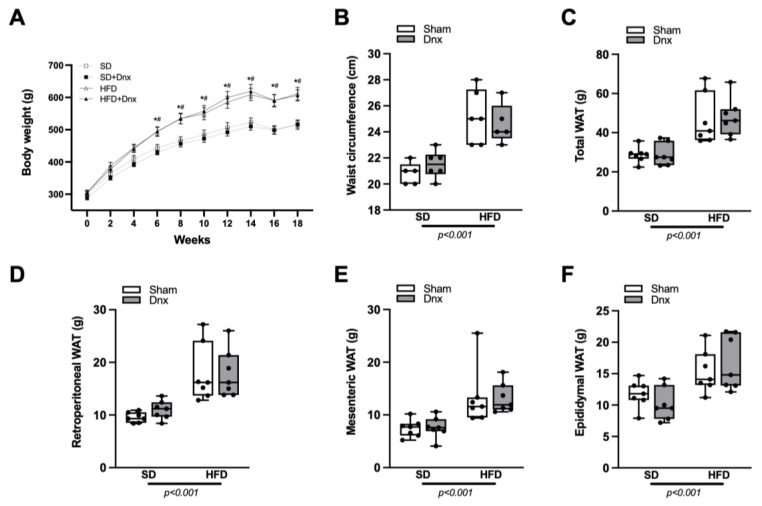
Body weight, waist circumference and white adipose tissue (WAT) pads (median and interquartile range) under standard diet (SD) or high-fat diet (HFD) in Sham groups (open bars) and Dnx (closed bars): (**A**) body weight, (**B**) waist circumference, (**C**) total WAT weight, (**D**) retroperitoneal WAT weight, (**E**) mesenteric WAT weight, (**F**) epididymal WAT weight. (**C**,**E**) Values were log-transformed to stabilize variance. A significant effect of diet was observed in (**B**) through (**F**). * *p >* 0.05 vs. SD; ^#^ *p* > 0.05 vs SD+Dnx. N = 7 each group.

At baseline, BW did not differ among the four groups, but after six weeks until the end of the 18th week, HFD and HFD+Dnx groups presented statistically higher BW compared to the standard diet (SD)-corresponding groups, SD and SD+Dnx (Figure 3A). At the end of the protocol, WC, total WAT, retroperitoneal WAT, mesenteric WAT and epididymal WAT pad weights were significantly higher in HFD groups than SD groups, but no effect was observed after Dnx (Figure 3B–F). Both Dnx groups presented significantly lower tyrosine hydroxylase protein levels in rWAT compared to both Sham groups, testifying that Dnx surgery had been successful (Supplementary Figure S1).

### 3.2. Bone Histomorphometric Parameters before and after Dnx

**Figure 4:**
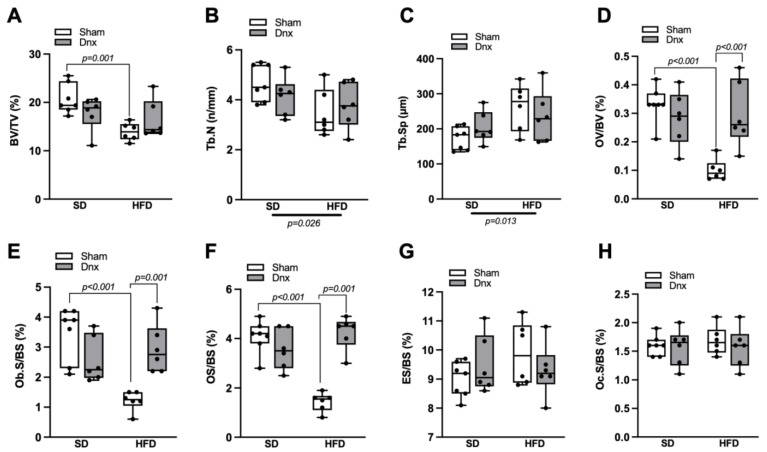
Static histomorphometric parameters (median and interquartile range) under standard diet (SD) or high fat diet (HFD) in Sham groups (open bars) and Dnx groups (closed bars): (**A**) bone volume—BV/TV, (**B**) trabecular number—Tb.N, (**C**) trabecular separation—Tb.Sp, (**D**) osteoid volume—OV/BV, (**E**) osteoblastic surface—Ob.S/BS, (**F**) osteoid surface—OS/BS, (**G**) eroded surface—ES/BS and (**H**) osteoclastic surface—Oc.S/BS. A significant effect of diet was observed in (**B**,**C**). Interaction was significant in (**A**) *p* = 0.044, (**D**) *p* = 0.001, (**E**) *p* < 0.001 and (**F**) *p* < 0.001. N = 6 each group.

The static bone histomorphometric parameters revealed that the HFD group presented significantly lower median values of trabecular volume (BV/TV), osteoid volume (OV/BV), osteoblastic surface (Ob.S/BS) and osteoid surface (OS/BS) than the SD group (Figure 4A,D–F). Additionally, significantly lower trabecular number (Tb.N) and higher trabecular separation (Tb.Sp) were observed in both HFD groups compared to both SD groups (Figure 4B,C). When compared to HFD, the HFD+Dnx group presented higher osteoid volume (OV/BV), osteoblastic surface (Ob.S/BS) and osteoid surface (OS/BS) (Figure 4D–F). No statistical differences in the remaining structural parameters such as trabecular thickness (Tb.Th) and osteoid thickness (O.Th) and resorption parameters like eroded surface (ES/BS) and osteoclastic surface (Oc.S/BS) were observed (Figure S2 and Figure 4G,H).

**Figure 5:**
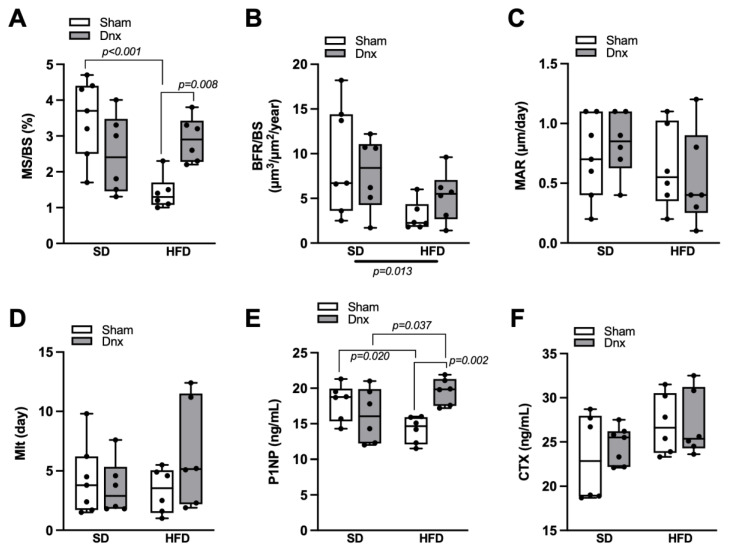
Dynamic histomorphometric parameters and serum bone formation and resorption markers (median and interquartile range) under standard diet (SD) or high fat diet (HFD) in Sham groups (open bars) and Dnx (closed bars): (**A**) mineralizing surface—MS/BS, (**B**) bone formation rate—BFR/BS, (**C**) mineral apposition rate—MAR and (**D**) mineralization lag time—Mlt. Serum (**E**) P1NP and serum (**F**) CTX. (**F**) values were log-transformed to stabilize variance. A significant effect of diet was observed in (**B**). Interaction was significant in (**A**) *p* = 0.002 and (**E**) *p* = 0.003. N = 6 each group.

Regarding dynamic bone histomorphometric parameters, HFD group presented significantly lower mineralizing surface (MS/BS) than SD group (Figure 5A), and bone formation rate (BFR/BS) was significantly lower in both HFD and HFD+Dnx versus their SD counterparts (Figure 5B). Moreover, HFD+Dnx presented significantly higher MS/BS when compared to HFD (Figure 5A). The remaining parameters such as mineral apposition rate (MAR) and mineralization lag time (Mlt) were not statistically different among all groups (Figure 5C,D).

### 3.3. Serum Bone Markers before and after Dnx

Serum P1NP was significantly lower in HFD compared to SD, but significantly higher in HFD+Dnx compared to HFD and SD+Dnx groups (Figure 5E). Serum CTX was not statistically different among all groups (Figure 5F).

### 3.4. Serum and Bone Hormones and Biochemical Markers before and after Dnx

**Figure 6:**
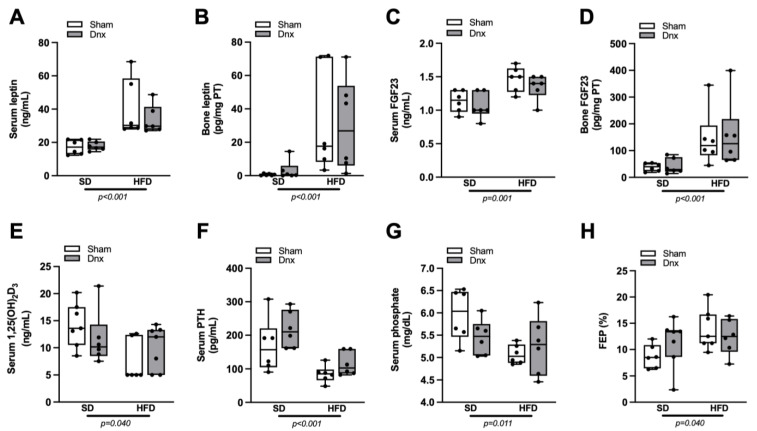
Serum calciotropic hormones/levels of serum and urinary phosphate/bone levels of leptin and FGF23 (median and interquartile range) under standard diet (SD) or high fat diet (HFD) in Sham groups (open bars) and Dnx (closed bars): (**A**) serum leptin, (**B**) bone leptin protein expression, (**C**) serum FGF23, (**D**) bone FGF23 protein expression, (**E**) serum 1,25(OH)_2_D_3_, (**F**) serum PTH, (**G**) serum phosphate and (**H**) fractional excretion of phosphate—FEP. (**B**,**D**) Values were log-transformed to stabilize variance. A significant effect of diet was observed in (**A**) through (**H**). N = 6 each group.

**Figure 7:**
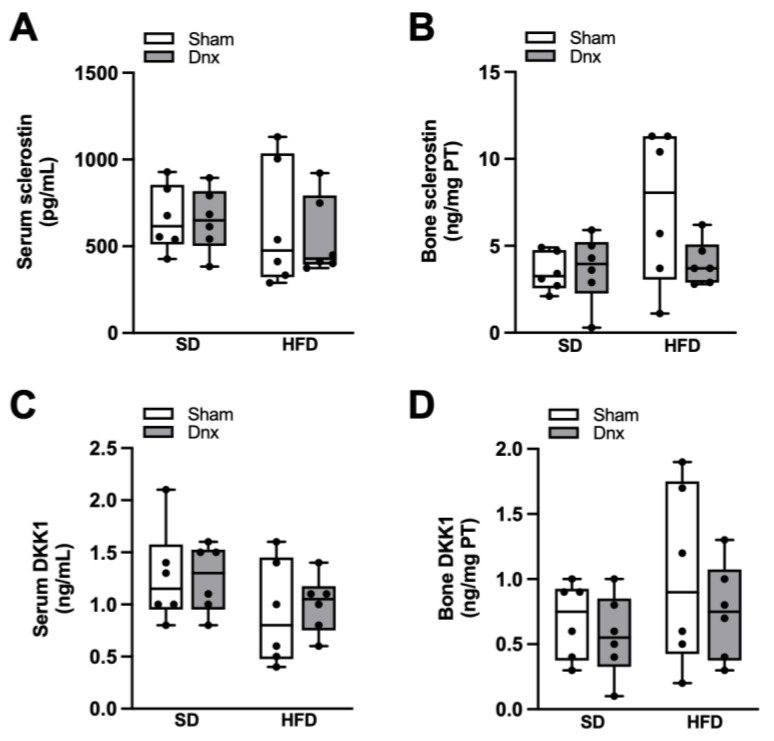
Serum and bone levels of sclerostin/DKK1 (median and interquartile range) under standard diet (SD) or high fat diet (HFD) in Sham groups (open bars) and Dnx (closed bars): (**A**) serum sclerostin, (**B**) bone sclerostin, (**C**) serum DKK1 and (**D**) bone DKK1. (**B**,**D**) Values were log-transformed to stabilize variance. N = 6 each group.

Significantly higher serum and bone levels of leptin and FGF23, higher FEP and lower serum 1,25(OH)_2_D_3_, PTH and phosphate were observed in both HFD groups compared to both SD groups while no effect was depicted after Dnx (Figure 6A–H). There were no statistically significant changes observed in serum calcium, FECa and 25(OH)D_3_ levels (Supplementary Figure S3). Serum levels and bone expression of sclerostin and DKK1 levels also did not differ among groups (Figure 7A–D).

### 3.5. Serum and Hypothalamic NPY Levels before and after Dnx

**Figure 8:**
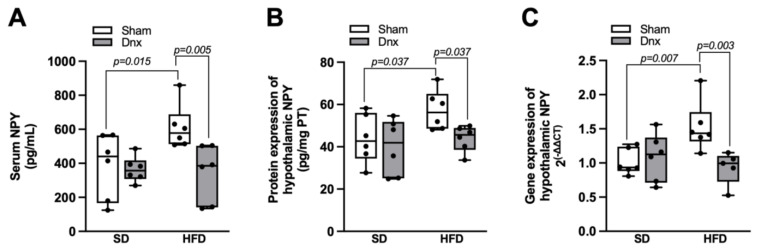
Serum levels and hypothalamic protein and gene expression of NPY (median and interquartile range) under standard diet (SD) or high fat diet (HFD) in Sham groups (open bars) and Dnx (closed bars): (**A**) serum NPY, (**B**) hypothalamic NPY protein levels and (**C**) hypothalamic NPY gene expression. Interaction was significant in (**A**) *p* < 0.001, (**B**) *p* < 0.001 and (**C**) *p* = 0.014. N = 6 each group.

Serum levels of NPY and hypothalamic NPY protein and gene expression were significantly higher in HFD compared to SD group. However, all these parameters were significantly decreased in HFD+Dnx compared to HFD (Figure 8A–C).

## 4. Discussion

The relationship between obesity and bone mass is complex and still debatable. The increase in adipose afferent reflex observed in obesity is known to induce sympathetic overactivity to different target organs such as heart, vessels, kidney and bone [27,33]. The current experimental model of rWAT denervation has been designed in order to address how the adipose tissue–CNS–bone signaling modulates changes in bone remodeling induced by obesity.

The present study disclosed that rWAT denervation blunted the decrease in bone formation observed in HFD-induced obesity rat model possibly through a preferential reduction in the neurohormonal actions of serum and hypothalamic NPY.

The reduced bone formation in HFD animals observed in this study evidenced by both decreased serum P1NP and histomorphometric bone formation parameters (lower Tb.N, higher Tb.Sp and consequent lower BV/TV, accompanied by lower BFR/BS in tibia) is in agreement with previous observations from Tencerova et al. [34] in a model of HFD-induced obesity in mice. Accordingly, current findings might have been ascribed to a shift favoring differentiation of bone marrow mesenchymal stem cells (BM-MSC) into adipocytes, at the expense of compromised osteoblast differentiation and bone formation [35]. The lack of alterations in histomorphometric indices of bone resorption as well as in serum markers such as CTX in HFD groups in the current HFD-induced obesity animal model is in line with other investigators [34] although differing from other obesity models in which metabolic syndrome had been induced by high carbohydrate levels, resulting in higher eroded surface and CTX levels [36]. The effects of leptin on bone cells are known to be site-specific [14]. Although the increased sympathetic tone through hypothalamic relay is expected to inhibit bone formation and increase bone resorption, opposite effects have been described at peripheral levels acting directly on osteoblasts, leading to reduced bone resorption and increased bone formation [14,16,17].

The increased levels of serum leptin in HFD rats found herein are in line with a recent study using a similar HFD-induced obesity design [27] and importantly, the current observation of higher levels of leptin in bone tissue further emphasizes the impact of adiposity in bone remodeling. Both positive and negative associations of circulating leptin with bone mass have already been reported in clinical and experimental studies [14,37,38,39,40] supporting the persistent controversy. Here, we also observed increased serum and bone protein levels of FGF23 in HFD rats, coupled with decreased serum levels of 1,25(OH)_2_D_3_ and phosphate and higher fractional excretion of phosphate, the latter effect as an expected coordinated modulation of renal phosphate handling by FGF23 [41]. Such findings agree well with Tsuji et al. [42] who suggested that leptin directly stimulates FGF-23 bone production inhibiting 1,25(OH)_2_D_3_ synthesis. The present high FGF23 levels in the setting of reduced bone formation found in histomorphometry in addition to lower P1NP, are coherent with in vitro observations showing suppression of osteoblast differentiation and matrix mineralization induced by FGF23 overexpression [43]. The decreased levels of PTH observed in HFD rats might be ascribed to the direct actions of FGF23 on the parathyroid through the MAPK pathway [44]. Unexpectedly, despite reduced bone formation in HFD rats, reductions in serum and bone protein levels of sclerostin and DKK1 were not evidenced in the present study, for reasons that remain unclear.

Finally, we observed higher levels of serum and hypothalamic NPY in HFD rats, corroborating clinical and experimental data in obesity conditions [45,46]. Moreover, NPY knockout mice present increased bone mass resulting from enhanced osteoblast activation and conversely, hypothalamic NPY overexpression reduces osteoblastic activity [25], suggesting a critical role of such mediators controlling bone formation. In a clinical study of patients with chronic kidney disease, Panuccio et al. [26] also demonstrated inverse associations of NPY levels with alkaline phosphatase reflecting osteoblast activity. In agreement with our present findings related to bone resorption, Matic et al. [47] did not observe increased osteoclast activity in mice overexpressing NPY.

Experimental evidence in mice demonstrates that, at the hypothalamic arcuate nucleus, the leptin receptor is co-expressed with NPY-positive neurons and its activation inhibits NPY secretion, promoting anorexigenic effects [48]. However, prolonged exposure to increased leptin levels as observed in obese subjects may promote leptin resistance leading to a disruption in the regulation of NPY secretion by leptin and consequently imbalance in energy homeostasis [49].

To the best of our knowledge, this is the first study to show that bilateral removal of rWAT innervation blunts the decrease in bone formation parameters in obese animals, namely Ob.S/BS, OV/BV, OS/BS, bone formation marker P1NP and mineralization surface (MS/BS), without changing bone resorption, as shown by histomorphometry and lack of alterations in CTX. It can be hypothesized that additional effects upon bone resorption after Dnx were not found because of the counteracting peripheral effects of leptin possibly reducing bone resorption that, in its turn, was not increased by obesity alone.

The higher serum leptin levels observed in HFD rats were not blunted after denervation surgery, contrasting with the findings of Garcia et al. [27]. However, in an elegant experimental study, Yamada et al. [50] showed that epididymal WAT denervation promoted a decrease in NPY expression in the hypothalamus without changes in serum glycaemia, insulin and leptin levels, suggesting that neuronal signals from intra-abdominal adipose tissue possibly participate in the NPY hypothalamic expression control, but that leptin regulation is independent of this signaling pathway. Moreover, we did not observe significant alterations in serum and bone FGF23 levels, nor in 1,25(OH)_2_D_3_ and PTH after Dnx surgery, suggesting that the latter did not change the peripheral signaling of these hormone secretions.

**Figure 9:**
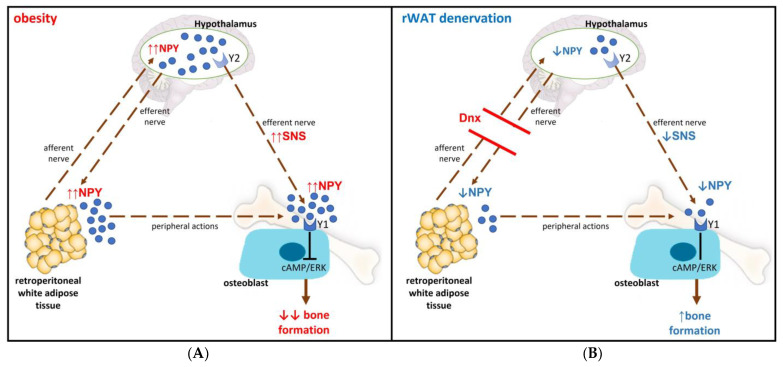
Hypothetical mechanism of the interaction between neuropeptide Y (NPY) and bone remodeling. (**A**) Obesity: increases in circulating and hypothalamic NPY levels may promote sympathetic overactivity to bone tissue through activation of hypothalamic Y2 receptor. NPY acts on Y1 receptor expressed in osteoblasts inhibiting the cAMP signaling pathway, followed by phosphorylation of ERK, which leads to reduced osteoblast differentiation and bone formation. (**B**) Retroperitoneal white adipose tissue (rWAT) denervation (Dnx): the interruption of the communication between rWAT and hypothalamus by Dnx might have restored central NPY levels resulting in normalization of sympathetic activation to rWAT and possibly to bone tissue as well, rescuing bone formation. Decreased peripheral levels of NPY induced by Dnx might have also contributed to restore bone formation. SNS—sympathetic nervous system.

In summary, the interruption of the communication between rWAT and the hypothalamus by the denervation procedure might have decreased hypothalamic NPY expression through an unknown mechanism resulting in decreased sympathetic activation to rWAT and possibly to bone tissue (hypothetical mechanism is shown in Figure 9).

Limitations of the present study included the lack of bone marrow gene expression evaluation and measurement of bone marrow adiposity. Since only male rats were used to minimize the confounding effects of female sex hormones on bone, the present findings cannot be extrapolated to female rats. Additional functional studies employing inhibitors of NPY receptors are still warranted to validate present findings and to further elucidate underlying cause–effect mechanisms between NPY and bone formation in the current model. Whether NPY actions in response to adipose tissue denervation are central, peripheral, or both, also remains to be investigated.

## 5. Conclusions

In conclusion, the present study demonstrated that rWAT denervation surgery blunted the negative effects of obesity on bone formation possibly through a preferential modulation by central or peripheral neurohormonal NPY actions, emphasizing the relevance of the adipose tissue–brain–bone axis in the control of bone metabolism in obesity.

## Data Availability

The data that support the findings of this study are available from the corresponding author upon reasonable request.

## Supplementary Materials

The following supporting information can be downloaded at: https://www.mdpi.com/article/10.3390/nu15163574/s1, Figure S1. Static histomorphometric parameters in Sham or denervated (Dnx) groups under standard diet (SD) or high fat diet (HFD). Figure S2. HFD did not alter calcium metabolism. Figure S3. Denervation surgery confirmation. Table S1. Composition of the diets used in the study. Table S2. List of antibodies used for Western Blot. Table S3. Primer sequences used for qPCR.

## References

[1] Tang X., Liu G., Kang J., Hou Y., Jiang F., Yuan W., Shi J. (2013). Obesity and risk of hip fracture in adults: A meta-analysis of prospective cohort studies. PLoS ONE.

[2] Johansson H., Kanis J.A., Odén A., McCloskey E., Chapurlat R.D., Christiansen C., Cummings S.R., Diez-Perez A., Eisman J.A., Fujiwara S. (2014). A meta-analysis of the association of fracture risk and body mass index in women. J. Bone Miner. Res..

[3] Qiao D., Li Y., Liu X., Zhang X., Qian X., Zhang H., Zhang G., Wang C. (2020). Association of obesity with bone mineral density and osteoporosis in adults: A systematic review and meta-analysis. Public Health.

[4] Ho-Pham L.T., Nguyen U.D., Nguyen T.V. (2014). Association between lean mass, fat mass, and bone mineral density: A meta-analysis. J. Clin. Endocrinol. Metab..

[5] Sutter T., Toumi H., Valery A., El Hage R., Pinti A., Lespessailles E. (2019). Relationships between muscle mass, strength and regional bone mineral density in young men. PLoS ONE.

[6] Tencerova M., Frost M., Figeac F., Nielsen T.K., Ali D., Lauterlein J.L., Andersen T.L., Haakonsson A.K., Rauch A., Madsen J.S. (2019). Obesity-Associated Hypermetabolism and Accelerated Senescence of Bone Marrow Stromal Stem Cells Suggest a Potential Mechanism for Bone Fragility. Cell Rep..

[7] Cohen A., Dempster D.W., Recker R.R., Lappe J.M., Zhou H., Zwahlen A., Müller R., Zhao B., Guo X., Lang T. (2013). Abdominal fat is associated with lower bone formation and inferior bone quality in healthy premenopausal women: A transiliac bone biopsy study. J. Clin. Endocrinol. Metab..

[8] Kuipers E.N., Held N.M., In Het Panhuis W., Modder M., Ruppert P.M.M., Kersten S., Kooijman S., Guigas B., Houtkooper R.H., Rensen P.C.N. (2019). A single day of high-fat diet feeding induces lipid accumulation and insulin resistance in brown adipose tissue in mice. Am. J. Physiol. Endocrinol. Metab..

[9] Maharjan B.R., McLennan S.V., Yee C., Twigg S.M., Williams P.F. (2021). The Effect of a Sustained High-Fat Diet on the Metabolism of White and Brown Adipose Tissue and Its Impact on Insulin Resistance: A Selected Time Point Cross-Sectional Study. Int. J. Mol. Sci..

[10] Grassi G., Biffi A., Seravalle G., Trevano F.Q., Dell’Oro R., Corrao G., Mancia G. (2019). Sympathetic Neural Overdrive in the Obese and Overweight State. Hypertension.

[11] Schlienger R.G., Kraenzlin M.E., Jick S.S., Meier C.R. (2004). Use of beta-blockers and risk of fractures. JAMA.

[12] Turker S., Karatosun V., Gunal I. (2006). Beta-blockers increase bone mineral density. Clin. Orthop. Relat. Res..

[13] Takeda S., Elefteriou F., Levasseur R., Liu X., Zhao L., Parker K.L., Armstrong D., Ducy P., Karsenty G. (2002). Leptin regulates bone formation via the sympathetic nervous system. Cell.

[14] Reid I.R., Baldock P.A., Cornish J. (2018). Effects of Leptin on the Skeleton. Endocr. Rev..

[15] Tamasi J.A., Arey B.J., Bertolini D.R., Feyen J.H. (2003). Characterization of bone structure in leptin receptor-deficient Zucker (fa/fa) rats. J. Bone Miner. Res..

[16] Cornish J., Callon K.E., Bava U., Lin C., Naot D., Hill B.L., Grey A.B., Broom N., Myers D.E., Nicholson G.C. (2002). Leptin directly regulates bone cell function in vitro and reduces bone fragility in vivo. J. Endocrinol..

[17] Goulding A., Taylor R.W. (1998). Plasma leptin values in relation to bone mass and density and to dynamic biochemical markers of bone resorption and formation in postmenopausal women. Calcif. Tissue Int..

[18] Dimitri P., Rosen C. (2017). The Central Nervous System and Bone Metabolism: An Evolving Story. Calcif. Tissue Int..

[19] Ekblad E., Edvinsson L., Wahlestedt C., Uddman R., Håkanson R., Sundler F. (1984). Neuropeptide Y co-exists and co-operates with noradrenaline in perivascular nerve fibers. Regul. Pept..

[20] Zhang Y., Chen C.Y., Liu Y.W., Rao S.S., Tan Y.J., Qian Y.X., Xia K., Huang J., Liu X.X., Hong C.G. (2021). Neuronal Induction of Bone-Fat Imbalance through Osteocyte Neuropeptide Y. Adv. Sci..

[21] Zoccali C., Ortiz A., Blumbyte I.A., Rudolf S., Beck-Sickinger A.G., Malyszko J., Spasovski G., Carriazo S., Viggiano D., Kurganaite J. (2021). Neuropeptide Y as a risk factor for cardiorenal disease and cognitive dysfunction in chronic kidney disease: Translational opportunities and challenges. Nephrol. Dial. Transpl..

[22] Lee N.J., Doyle K.L., Sainsbury A., Enriquez R.F., Hort Y.J., Riepler S.J., Baldock P.A., Herzog H. (2010). Critical role for Y1 receptors in mesenchymal progenitor cell differentiation and osteoblast activity. J. Bone Miner. Res..

[23] Parker R.M., Herzog H. (1999). Regional distribution of Y-receptor subtype mRNAs in rat brain. Eur. J. Neurosci..

[24] Yu W., Zhu C., Xu W., Jiang L., Jiang S. (2016). Neuropeptide Y1 Receptor Regulates Glucocorticoid-Induced Inhibition of Osteoblast Differentiation in Murine MC3T3-E1 Cells via ERK Signaling. Int. J. Mol. Sci..

[25] Baldock P.A., Lee N.J., Driessler F., Lin S., Allison S., Stehrer B., Lin E.J., Zhang L., Enriquez R.F., Wong I.P. (2009). Neuropeptide Y knockout mice reveal a central role of NPY in the coordination of bone mass to body weight. PLoS ONE.

[26] Panuccio V., Cutrupi S., Pizzini P., Mallamaci F., Tripepi G., Zoccali C. (2007). Neuropeptide Y and markers of osteoblast activity in dialysis patients: A cross-sectional study. Am. J. Kidney Dis..

[27] Garcia M.L., Milanez M.I.O., Nishi E.E., Sato A.Y.S., Carvalho P.M., Nogueira F.N., Campos R.R., Oyama L.M., Bergamaschi C.T. (2021). Retroperitoneal adipose tissue denervation improves cardiometabolic and autonomic dysfunction in a high fat diet model. Life Sci..

[28] Reeves P.G., Nielsen F.H., Fahey G.C. (1993). AIN-93 purified diets for laboratory rodents: Final report of the American Institute of Nutrition ad hoc writing committee on the reformulation of the AIN-76A rodent diet. J. Nutr..

[29] Frasson D., Boschini R.P., Chaves V.E., dos Santos M.E.S.M., Paula Gomes S., Valentim R.R., Garófalo M.A.R., Navegantes L.C.C., Migliorini R.H., Kettelhut I.C. (2012). The sympathetic nervous system regulates the three glycerol-3P generation pathways in white adipose tissue of fasted, diabetic and high-protein diet-fed rats. Metabolism.

[30] Gouveia C.H., Jorgetti V., Bianco A.C. (1997). Effects of thyroid hormone administration and estrogen deficiency on bone mass of female rats. J. Bone Miner. Res..

[31] Dempster D.W., Compston J.E., Drezner M.K., Glorieux F.H., Kanis J.A., Malluche H., Meunier P.J., Ott S.M., Recker R.R., Parfitt A.M. (2013). Standardized nomenclature, symbols, and units for bone histomorphometry: A 2012 update of the report of the ASBMR Histomorphometry Nomenclature Committee. J. Bone Miner. Res. Off. J. Am. Soc. Bone Miner. Res..

[32] Nishi E.E., Lopes N.R., Gomes G.N., Perry J.C., Sato A.Y.S., Naffah-Mazzacoratti M.G., Bergamaschi C.T., Campos R.R. (2019). Renal denervation reduces sympathetic overactivation, brain oxidative stress, and renal injury in rats with renovascular hypertension independent of its effects on reducing blood pressure. Hypertens. Res..

[33] Lambert E., Phillips S., Tursunalieva A., Eikelis N., Sari C., Dixon J., Straznicky N., Grima M., Schlaich M., Lambert G. (2018). Inverse association between sympathetic nervous system activity and bone mass in middle aged overweight individuals. Bone.

[34] Tencerova M., Figeac F., Ditzel N., Taipaleenmaki H., Nielsen T.K., Kassem M. (2018). High-Fat Diet-Induced Obesity Promotes Expansion of Bone Marrow Adipose Tissue and Impairs Skeletal Stem Cell Functions in Mice. J. Bone Miner. Res..

[35] Boroumand P., Klip A. (2020). Bone marrow adipose cells—Cellular interactions and changes with obesity. J. Cell Sci..

[36] Wong S.K., Chin K.Y., Suhaimi F.H., Ahmad F., Ima-Nirwana S. (2018). Effects of metabolic syndrome on bone mineral density, histomorphometry and remodelling markers in male rats. PLoS ONE.

[37] Kontogianni M.D., Dafni U.G., Routsias J.G., Skopouli F.N. (2004). Blood leptin and adiponectin as possible mediators of the relation between fat mass and BMD in perimenopausal women. J. Bone Miner. Res..

[38] Ducy P., Amling M., Takeda S., Priemel M., Schilling A.F., Beil F.T., Shen J., Vinson C., Rueger J.M., Karsenty G. (2000). Leptin inhibits bone formation through a hypothalamic relay: A central control of bone mass. Cell.

[39] Vilaca T., Evans A., Gossiel F., Paggiosi M., Eastell R., Walsh J.S. (2022). Fat, adipokines, bone structure and bone regulatory factors associations in obesity. Eur. J. Endocrinol..

[40] Pasco J.A., Henry M.J., Kotowicz M.A., Collier G.R., Ball M.J., Ugoni A.M., Nicholson G.C. (2001). Serum leptin levels are associated with bone mass in nonobese women. J. Clin. Endocrinol. Metab..

[41] Vervloet M. (2019). Renal and extrarenal effects of fibroblast growth factor 23. Nat. Rev. Nephrol..

[42] Tsuji K., Maeda T., Kawane T., Matsunuma A., Horiuchi N. (2010). Leptin stimulates fibroblast growth factor 23 expression in bone and suppresses renal 1alpha,25-dihydroxyvitamin D3 synthesis in leptin-deficient mice. J. Bone Miner. Res. Off. J. Am. Soc. Bone Miner. Res..

[43] Wang H., Yoshiko Y., Yamamoto R., Minamizaki T., Kozai K., Tanne K., Aubin J.E., Maeda N. (2008). Overexpression of fibroblast growth factor 23 suppresses osteoblast differentiation and matrix mineralization in vitro. J. Bone Miner. Res..

[44] Ben-Dov I.Z., Galitzer H., Lavi-Moshayoff V., Goetz R., Kuro-o M., Mohammadi M., Sirkis R., Naveh-Many T., Silver J. (2007). The parathyroid is a target organ for FGF23 in rats. J. Clin. Investig..

[45] Baltazi M., Katsiki N., Savopoulos C., Iliadis F., Koliakos G., Hatzitolios A.I. (2011). Plasma neuropeptide Y (NPY) and alpha-melanocyte stimulating hormone (a-MSH) levels in patients with or without hypertension and/or obesity: A pilot study. Am. J. Cardiovasc. Dis..

[46] Kuo L.E., Kitlinska J.B., Tilan J.U., Li L., Baker S.B., Johnson M.D., Lee E.W., Burnett M.S., Fricke S.T., Kvetnansky R. (2007). Neuropeptide Y acts directly in the periphery on fat tissue and mediates stress-induced obesity and metabolic syndrome. Nat. Med..

[47] Matic I., Matthews B.G., Kizivat T., Igwe J.C., Marijanovic I., Ruohonen S.T., Savontaus E., Adams D.J., Kalajzic I. (2012). Bone-specific overexpression of NPY modulates osteogenesis. J. Musculoskelet. Neuronal Interact..

[48] Dhillon S.S., Belsham D.D. (2011). Leptin differentially regulates NPY secretion in hypothalamic cell lines through distinct intracellular signal transduction pathways. Regul. Pept..

[49] Dhillon S.S., McFadden S.A., Chalmers J.A., Centeno M.L., Kim G.L., Belsham D.D. (2011). Cellular leptin resistance impairs the leptin-mediated suppression of neuropeptide Y secretion in hypothalamic neurons. Endocrinology.

[50] Yamada T., Katagiri H., Ishigaki Y., Ogihara T., Imai J., Uno K., Hasegawa Y., Gao J., Ishihara H., Niijima A. (2006). Signals from intra-abdominal fat modulate insulin and leptin sensitivity through different mechanisms: Neuronal involvement in food-intake regulation. Cell Metab..

