# Supplementary material for: Adipose Tissue Denervation Blunted the Decrease in Bone Formation Promoted by Obesity in Rats
Source: Nutrients. 2023 Aug 14;15(16):3574. doi: 10.3390/nu15163574 (PMC10458609; doi:10.3390/nu15163574)
Supplement: Supplementary file 1 [file nutrients-15-03574-s001.zip › nutrients-2525209-supplementary.pdf]

nutrients

Article

Adipose tissue denervation blunted the decrease in bone formation promoted by obesity in rats

Milene Subtil Ormanji[1], Maria Victória Lazarini Melo[1], Renata Meca[1], Michelle Louvaes Garcia[2], Ana Carolina Anauate[1], Juan José Augusto Moyano Muñoz[1], Lila Missae Oyama[2], Erika Emy Nishi[2], Cassia T. Bergamaschi[2], Aluizio Barbosa Carvalho[1], Ita Pfeferman Heilberg[1*]

[1] Nephrology Division, Universidade Federal de São Paulo, 04023-062, São Paulo, Brazil milene.ormanji@gmail.com (M.S.O.); mavi.lazarini@hotmail.com (M.V.L.M.) rmeca28@gmail.com (R.M.); anauatte@gmail.com (A.C.A.); juanjomoy@gmail.com (J.J.A.M.M.); aluizio@uol.com.br (A.B.C.)
[2] Department of Physiology, Universidade Federal de São Paulo, 04023-062, São Paulo, Brazil michelle.louvaes@gmail.com (M.L.G.); lmoyama@unifesp.br (L.M.O.); enishi@unifesp.br (E.E.N.); bergamaschi.cassia@unifesp.br (C.T.B.)
* Correspondence: ita.heilberg@gmail.com (I.P.H.); Tel.: +55(11) 5576-4848 (ext 2465)

SUPPLEMENTARY FIGURES

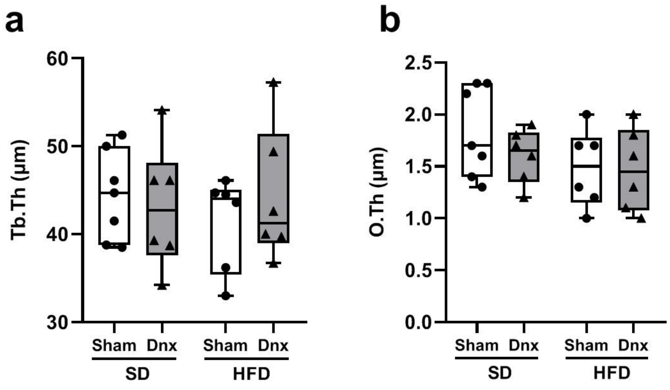

Figure S1. Static histomorphometric parameters in Sham or denervated (Dnx) groups under standard diet (SD) or high fat diet (HFD). (a) trabecular thickness – Tb.Th, (b) osteoid thickness – O.Th. Open bars (Sham groups) and closed bars (Dnx) represent median and interquartile range. Statistical analyses were performed by two-way ANOVA with Bonferroni post-hoc test. P<0.05 was considered statistically significant.

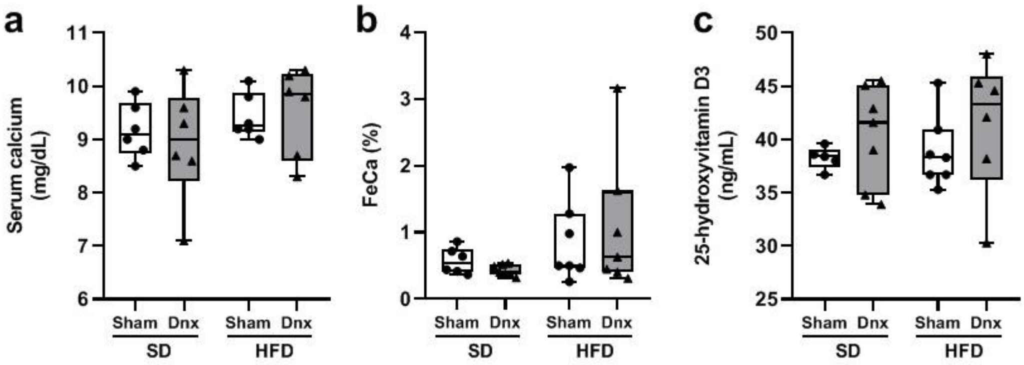

Figure S2. HFD did not alter calcium metabolism. Biochemical parameters evaluated at the end of protocol did not differ among groups: (a) serum calcium; (b) urinary fractional excretion of calcium – FECa (%); (c) serum 25-hydroxyvitamin D_3. Bars represent median and interquartile range. Statistical analyses were performed by two-way ANOVA with Bonferroni post-hoc test. P<0.05 was considered statistically significant.

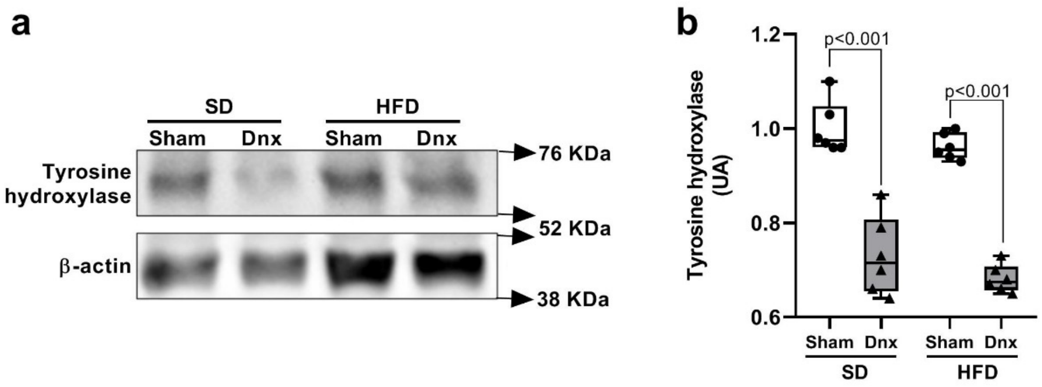

Figure S3. Denervation surgery confirmation. Denervation of retroperitoneal white adipose tissue (rWAT) was confirmed by western blot quantification of tyrosine hydroxylase protein, which was significantly lower in SD+Dnx and HFD+Dnx groups when compared to non-denervated groups (Sham SD and HFD). (a) representative western blots of relative protein levels and (b) relative quantification of tyrosine hydroxylase protein. Bars represent median and interquartile range. Statistical analyses were performed by two-way ANOVA with Bonferroni post-hoc test. P<0.05 was considered statistically significant.

SUPPLEMENTARY TABLES

Table S1. Composition of the diets used in the study.

**Ingredients (g/kg of diet)**	**SD**	**HFD**
Cornstarch	720.7	248.7
Sucrose	-	100.0
Casein	140.0	200.0
Soybean oil	40.0	40.0
Cellulose	50.0	50.0
Lard	-	312.0
AIN-93M Vitamin mixture	10.0	10.0
AIN-93M Mineral mixture	35.0	35.0
L-cystine	1.8	1.8
Choline bitartrate	2.5	2.5
Butyl hydroquinone	0.008	0.008

SD: standard diet; HFD: high-fat diet

Table S2. List of antibodies used for Western Blot.

**Protein/Epitope**	**Source and Catalog number**	**Host**	**Application**	**Dilution**	**Application specific details**
Tyrosine hydroxylase	Cell Signaling, 2792S	rabbit	Western Blot	1:1000	5% BSA, overnight, 4°C
Anti-rabbit IgG	Abcam, ab97051	goat	Western Blot	1:50000	5% BSA, 1hr RT

Table S3. Primer sequences used for qPCR.

**Gene**	**Forward sequence**	**Reverse sequence**
NPY	5’ CAGATACTACTCCGCTCTGC 3’	5’ AAGGGTCTTCAAGCCTTGTT 3’
Gapdh	5’ CAACTCCCTCAAGATTGTCAGCAA 3’	5’ GGCATGGACTGTGGTCATGA 3’
